# Supplementary material for: Economic and social modulations of innate decision-making in mice exposed to visual threats
Source: eLife. 2026 Aug 18;14:RP107306. doi: 10.7554/eLife.107306 (PMC13485303; doi:10.7554/eLife.107306)
Supplement: Peer review history

# Editor report: eLife Assessment

**DOI:** 10.7554/eLife.107306.5.sa0

**Authors:** Alicia Izquierdo

**Affiliations:** University of California, Los Angeles United States

**Keywords:** Solid, Valuable

The authors show that innate defensive behavior in mice is shaped by threat intensity, reward value, and social hierarchy, highlighting how value and social context influence instinctive decisions. The authors provide a **valuable** characterization of escape behavior which approximates naturalistic conditions. Despite minor methodological limitations, the work provides a **solid** foundation for future investigation of how reward and social context interact to influence behavior.

# Referee report: Reviewer #1 (Public review)

**DOI:** 10.7554/eLife.107306.5.sa1

[Editors' note: this version has been assessed by the Reviewing Editor without further input from the original reviewers. The authors have addressed the comments in their discussion of the limitations that were raised in the previous round of review.]

This study by Li and colleagues examines how defensive responses to visual threats during foraging are modulated by both reward level and social hierarchy. Using a semi-naturalistic paradigm, the authors test how the availability of water or sucrose, with sucrose being more rewarding than water, shapes escape behavior in mice exposed to looming stimuli of different intensities, which are used to probe perceived threat level and defensive responses. In parallel, the study compares dominant and subordinate animals to assess how social rank biases the trade-off between reward seeking and threat avoidance. By combining behavioral analyses with computational modeling, the work addresses how reward level and social context jointly influence escape decisions in an ethological setting.

Across the different experimental conditions, perceived threat level is the main determinant of behavior. The authors show that looming stimuli associated with higher threat (contrast) consistently elicit faster and more robust escape responses than lower threat stimuli. This effect is particularly evident during early exposures, when animals are highly vigilant and have not yet habituated to the looming stimulus (learned that it is not dangerous). Later they described that as animals gain experience and habituate, behavior becomes more flexible, and reward level begins to exert a graded modulation of the escape response. Importantly, the authors show that under high threat conditions increasing reward value leads to more frequent and faster escape rather than greater reward pursuit, specifically in dominant mice. This finding is particularly relevant, as it suggests that highly valued rewards can heighten vigilance and thereby enhance responsiveness to threat, highlighting that reward does not simply compete with defensive behavior but can also reshape it depending on the perceived level of danger, in contrast to low threat conditions, where threat can be more easily outweighed by reward. However, it is worth noting that the authors use an extremely low contrast for the low threat condition (20%), which may to some extent be insufficient to reliably trigger escape responses. Thus, an important conceptual contribution of the study is the introduction of vigilance as a useful framework to interpret these effects. Vigilance is treated as a behavioral state reflecting heightened attention to potential danger. In line with what is known from natural foraging, mice initially maintain high vigilance when confronted with an innate threat. This perspective helps clarify a finding that might otherwise appear counterintuitive. One might expect higher rewards to motivate animals to tolerate risk, explore more, and habituate faster in any scenario. Instead, the data suggest that highly rewarding outcomes can elevate vigilance, making animals more responsive to threat and leading to faster or more frequent escape under high threat conditions. In this sense, reward does not simply compete with threat but can also amplify sensitivity to it, depending on the internal state of the animal.

The social results are particularly interesting in this context as well. Dominant mice consistently prioritize avoidance over reward, showing stronger escape responses and slower habituation than subordinates. This behavior is well captured by the vigilance framework proposed by the authors: dominant animals appear to maintain higher vigilance, which biases decisions toward threat avoidance. The authors further suggest that stable social relationships sustain high vigilance and slow habituation, framing this as an evolutionarily conserved strategy that may enhance survival. This interpretation provides a valuable perspective on how social structure shapes defensive behavior beyond immediate physical interactions. At the same time, there are important limitations to this interpretation. All experiments were conducted in male mice, and it is possible that the relationship between social hierarchy, vigilance, and defensive behavior would differ substantially in females. In addition, the idea that stable social relationships sustain elevated vigilance should be interpreted carefully, as it does not fully align with broader views of social stability as protective against anxiety and stress and generally beneficial for mental health and resilience. These points do not undermine the findings but suggest that the social effects described here should be interpreted with caution and within the specific context of the task and sex studied.

Another important limitation is that the neural mechanisms underlying these effects remain highly speculative. Although the manuscript includes an extensive discussion of candidate circuits, particularly involving the superior colliculus and downstream structures, these interpretations go far beyond the data presented in the study and are not directly supported by experimental evidence within the paper itself. The discussion gives substantial weight to potential circuit mechanisms based primarily on previous literature rather than on findings from the current study. Given the complexity and distributed nature of the circuits likely involved in integrating vigilance, reward, social context, and defensive behavior, the present work is better viewed as providing a strong behavioral framework rather than direct mechanistic insight into the underlying neural substrates. In this context, some references discussing how animals learn to suppress defensive responses to repeated looming threats and the neural mechanisms supporting this process could further strengthen the discussion (Salay et al 2021; Fratzl et al. 2021; Conway et al. 2025; Mederos et al. 2025).

Methodologically, the behavioral paradigm is well suited for studying escape decisions in socially housed animals, and the machine learning based classification of defensive responses is a strength. The computational model provides a useful formalization of how threat level, reward level, and vigilance interact and may be valuable for other laboratories studying escape, approach avoidance, or conflict situations, particularly as a way to classify behavioral outcomes after pose estimation. More generally, the work will be of interest to the neuroethology community for its detailed characterization of escape behavior under naturalistic conditions. At the same time, some statements in the discussion slightly overstate the novelty of the methodological approach. For example, the claim that the study differs from earlier work by using machine learning rather than manual annotation overlooks that several previous studies have already implemented automated or semi-automated strategies to classify looming evoked defensive behaviors beyond manual scoring alone.

Given the ethological nature of the study and the high inter individual variability reported by the authors, clarity and precision in the methods are especially important for reproducibility. While the revised manuscript addresses many earlier concerns, some aspects remain slightly difficult to follow. For example, the main text states that animals were not water deprived to minimize differences in internal state across conditions, whereas parts of the methods describe experiments in which animals were water deprived. This distinction is not always clearly explained across the different experimental sections, despite internal state being central to the interpretation of the behavioral findings. A clearer separation and description of these conditions would further strengthen confidence in the work. In addition, it was somewhat surprising that the low contrast (20%) looming condition was still sufficient to trigger robust escape responses, and additional clarification or discussion regarding stimulus saliency at this contrast level could help readers better contextualize these findings.

Overall, this study provides a rich analysis of how reward level and social hierarchy modulate defensive behavior through changes in vigilance. It offers a useful conceptual advance for thinking about escape behavior in semi-naturalistic settings and lays a solid foundation for future work aimed at linking these behavioral states to underlying neural circuits.

# Author comment: Author response

**DOI:** 10.7554/eLife.107306.5.sa2

**Authors:** Zhe Li, Jiahui Wang, Yidan Sun, Jialin Li, Ling-yun Li, Ya-tang Li

**Affiliations:** Chinese Institute for Brain Research Beijing China; Chinese Institute for Brain Research Beijing China; Chinese Institute for Brain Research Beijing China; Chinese Institute for Brain Research Beijing China; Capital Medical University Beijing China; Chinese Institute for Brain Research Beijing China

The following is the authors’ response to the previous reviews

**Public Reviews:**

**Reviewer #1 (Public review):**

This study by Li and colleagues examines how defensive responses to visual threats during foraging are modulated by both reward level and social hierarchy. Using a semi-naturalistic paradigm, the authors test how the availability of water or sucrose, with sucrose being more rewarding than water, shapes escape behavior in mice exposed to looming stimuli of different intensities, which are used to probe perceived threat level and defensive responses. In parallel, the study compares dominant and subordinate animals to assess how social rank biases the trade-off between reward seeking and threat avoidance. By combining behavioral analyses with computational modeling, the work addresses how reward level and social context jointly influence escape decisions in an ethological setting.

Across the different experimental conditions, perceived threat level is the main determinant of behavior. The authors show that looming stimuli associated with higher threat (contrast) consistently elicit faster and more robust escape responses than lower threat stimuli. This effect is particularly evident during early exposures, when animals are highly vigilant and have not yet habituated to the looming stimulus (learned that it is not dangerous). Later they described that as animals gain experience and habituate, behavior becomes more flexible, and reward level begins to exert a graded modulation of the escape response. Importantly, the authors show that under high threat conditions increasing reward value leads to more frequent and faster escape rather than greater reward pursuit, specifically in dominant mice. This finding is particularly relevant, as it suggests that highly valued rewards can heighten vigilance and thereby enhance responsiveness to threat, highlighting that reward does not simply compete with defensive behavior but can also reshape it depending on the perceived level of danger, in contrast to low threat conditions, where threat can be more easily outweighed by reward. However, it is worth noting that the authors use an extremely low contrast for the low threat condition (20%), which may to some extent be insufficient to reliably trigger escape responses. Thus, an important conceptual contribution of the study is the introduction of vigilance as a useful framework to interpret these effects. Vigilance is treated as a behavioral state reflecting heightened attention to potential danger. In line with what is known from natural foraging, mice initially maintain high vigilance when confronted with an innate threat. This perspective helps clarify a finding that might otherwise appear counterintuitive. One might expect higher rewards to motivate animals to tolerate risk, explore more, and habituate faster in any scenario. Instead, the data suggest that highly rewarding outcomes can elevate vigilance, making animals more responsive to threat and leading to faster or more frequent escape under high threat conditions. In this sense, reward does not simply compete with threat but can also amplify sensitivity to it, depending on the internal state of the animal.

We agree that the low-contrast condition (20%) represents a relatively weak threat signal by design. This level was intentionally chosen to fall within a regime that biases behavior toward freezing and escape after assessment rather than direct escape, allowing us to examine graded decision-making across threat intensities. The higher escape probability observed under low-contrast conditions relative to previous studies (Evans et al., 2018; Fratzl et al., 2021) is likely attributable to the linear arena used here, which generally promoted escape behavior over freezing.

The social results are particularly interesting in this context as well. Dominant mice consistently prioritize avoidance over reward, showing stronger escape responses and slower habituation than subordinates. This behavior is well captured by the vigilance framework proposed by the authors: dominant animals appear to maintain higher vigilance, which biases decisions toward threat avoidance. The authors further suggest that stable social relationships sustain high vigilance and slow habituation, framing this as an evolutionarily conserved strategy that may enhance survival. This interpretation provides a valuable perspective on how social structure shapes defensive behavior beyond immediate physical interactions. At the same time, there are important limitations to this interpretation. All experiments were conducted in male mice, and it is possible that the relationship between social hierarchy, vigilance, and defensive behavior would differ substantially in females. In addition, the idea that stable social relationships sustain elevated vigilance should be interpreted carefully, as it does not fully align with broader views of social stability as protective against anxiety and stress and generally beneficial for mental health and resilience. These points do not undermine the findings but suggest that the social effects described here should be interpreted with caution and within the specific context of the task and sex studied.

We thank the reviewer for this important comment and agree that findings obtained in male mice may not necessarily generalize to female mice. We have acknowledged the limitation in the Discussion and note that future studies will be required to determine the extent to which the present findings extend to female mice.

We would also like to clarify our interpretation regarding vigilance and social buffering. Vigilance should not be conflated with stress or anxiety, and the slower habituation observed in group-housed mice reflects sustained sensitivity to repeated threat exposure rather than elevated anxiety. Thus, our data do not contradict the concept of social buffering; rather, they are consistent with it, as pair-housed mice exhibited reduced defensive responses compared with individually housed animals (Lenz et al., 2022). Furthermore, in an independent study (Li, Gao, and Li, 2026; eLife 15:RP109571), we directly compared responses to looming stimuli when mice were tested alone versus in the presence of a social partner and found clear evidence of social buffering. These findings suggest that social interactions can attenuate defensive responses while prolonging vigilance during repeated threat exposure. We have revised the Discussion accordingly.

Another important limitation is that the neural mechanisms underlying these effects remain highly speculative. Although the manuscript includes an extensive discussion of candidate circuits, particularly involving the superior colliculus and downstream structures, these interpretations go far beyond the data presented in the study and are not directly supported by experimental evidence within the paper itself. The discussion gives substantial weight to potential circuit mechanisms based primarily on previous literature rather than on findings from the current study. Given the complexity and distributed nature of the circuits likely involved in integrating vigilance, reward, social context, and defensive behavior, the present work is better viewed as providing a strong behavioral framework rather than direct mechanistic insight into the underlying neural substrates. In this context, some references discussing how animals learn to suppress defensive responses to repeated looming threats and the neural mechanisms supporting this process could further strengthen the discussion (Salay et al 2021; Fratzl et al. 2021; Conway et al. 2025; Mederos et al. 2025).

We agree that the proposed neural mechanisms remain speculative and that the circuits involved in integrating internal state, reward, and social context are likely far more complex. We have revised the manuscript to acknowledge this limitation and have included a discussion of learning-dependent suppression of defensive responses to repeated looming threats and the underlying circuit mechanisms.

Methodologically, the behavioral paradigm is well suited for studying escape decisions in socially housed animals, and the machine learning based classification of defensive responses is a strength. The computational model provides a useful formalization of how threat level, reward level, and vigilance interact and may be valuable for other laboratories studying escape, approach avoidance, or conflict situations, particularly as a way to classify behavioral outcomes after pose estimation. More generally, the work will be of interest to the neuroethology community for its detailed characterization of escape behavior under naturalistic conditions. At the same time, some statements in the discussion slightly overstate the novelty of the methodological approach. For example, the claim that the study differs from earlier work by using machine learning rather than manual annotation overlooks that several previous studies have already implemented automated or semi-automated strategies to classify looming evoked defensive behaviors beyond manual scoring alone.

We have revised the manuscript to moderate our claims regarding the novelty of the machine-learning–based classification approach.

Given the ethological nature of the study and the high inter individual variability reported by the authors, clarity and precision in the methods are especially important for reproducibility. While the revised manuscript addresses many earlier concerns, some aspects remain slightly difficult to follow. For example, the main text states that animals were not water deprived to minimize differences in internal state across conditions, whereas parts of the methods describe experiments in which animals were water deprived. This distinction is not always clearly explained across the different experimental sections, despite internal state being central to the interpretation of the behavioral findings. A clearer separation and description of these conditions would further strengthen confidence in the work. In addition, it was somewhat surprising that the low contrast (20%) looming condition was still sufficient to trigger robust escape responses, and additional clarification or discussion regarding stimulus saliency at this contrast level could help readers better contextualize these findings.

To improve clarity, we have revised the Methods section to clearly distinguish between experimental conditions that involved water deprivation and those that did not.

Overall, this study provides a rich analysis of how reward level and social hierarchy modulate defensive behavior through changes in vigilance. It offers a useful conceptual advance for thinking about escape behavior in semi-naturalistic settings and lays a solid foundation for future work aimed at linking these behavioral states to underlying neural circuits.

**Recommendations for the authors:**

**Reviewer #1 (Recommendations for the authors):**

- Clarify which experiments involved water deprivation and which did not in methods section.

We have revised the Methods section to clearly specify which experiments involved water deprivation and which were conducted without water deprivation.

- Add discussion on why the 20% low contrast stimulus is still sufficient to trigger escape responses.

We have expanded the Discussion regarding the 20% contrast stimulus to clarify why it was sufficient to elicit escape responses.

- Tone down statements regarding the novelty of the machine learning based behavioral classification.

We have revised the manuscript to moderate claims regarding the novelty of the machine-learning–based classification approach.

- Include references related to learning-dependent suppression of looming responses and circuit mechanisms

We have incorporated the suggested references and expanded the Discussion of learning-dependent suppression of looming responses and the underlying circuit mechanisms.

- Moderate the discussion of candidate neural circuits, particularly the SC related interpretations, as these are not directly tested in the study.

We have revised the discussion of candidate circuit mechanisms.

- Clarify that the interpretation linking stable social relationships to elevated vigilance may be specific to this ethological context.

We have revised the Discussion to clarify this interpretation.
